# Enhancing the quality of elastane-cotton core yarn by compact spinning

**DOI:** 10.1016/j.heliyon.2022.e09562

**Published:** 2022-05-30

**Authors:** Md. Ikramul Islam, Ahmed Jalal Uddin

**Affiliations:** Department of Yarn Engineering, Bangladesh University of Textiles, Tejgaon, Dhaka, 1208, Bangladesh

**Keywords:** Core yarn, Elastane, Compact spinning, Conventional spinning, Morphologies and physical properties

## Abstract

The quest for highly stretchable fabrics with good aesthetic and functional properties has led researchers to constantly involve in mingling of natural and synthetic fibers. Elastane-cotton core yarns have an increasing demand due to their wear comfort and stretch-to-fit properties; therefore, efforts are still going on to optimize the yarn properties to meet the requirements for diversified applications. With a view to enhancing the appearance and performance characteristics of elastane-cotton core yarns, the present work was undertaken to manufacture them by exploiting the most modern and versatile pneumatic compacting mechanism, namely Suessen's EliTe compact spinning system. Elastane-cotton core yarns of different counts (20 tex, 30 tex and 60 tex) were produced with compact and conventional ring spinning system. The difference in morphology and physical properties of two types of yarns were compared after a thorough investigation by scanning electron microscope, evenness tester and strength tester. The results exhibited a noticeable decrease in hairiness, hairiness variation and neps values, especially seed-coat neps, for elastane-cotton compact core yarn. Unevenness & imperfection of compact core yarns were also found to be decreased that were reflected to the proportional enhancements in tenacity & elongation values. The overall observation reveals the potential of pneumatic compacting mechanism in obtaining elastane-cotton core yarn with superior structure and improved mechanical properties.

## 1 Introduction

Core-spun yarns are increasingly gained attention since their applications in the textile and industrial field have been diversified. These yarns were developed with a view to utilizing the properties of two or more fiber components at the same time (Qadir et al., 2014). The core yarns are usually composed of two different components; a sheath and a core. The core is generally a continuous monofilament or multifilament that imparts mechanical and functional properties, and cotton is mostly used as a sheath due to its commendable absorbance, comfort, feel and other unique properties that can hardly be found in any other fiber (Kılıç et al., 2020). Production of core yarns need a core-feed attachment that was found to be compatible with a number of spinning systems such as conventional ring spinning (Sarıoğlu and Babaarslan, 2019; Babaarslan, 2001), friction spinning (Merati et al., 1998) and air-jet spinning (Pramanik and Patil, 2009).

The usage of elastane-cotton core yarns is growing fast day by day to produce different kinds of stretch denim. Elastane-cotton core yarns comprise an elastane filament in the center encircled by cotton fibers. These yarns give materials the appropriate stretch and recovery without causing undesirable deformation over service life of garments (Şengöz, 2004; Zhang et al., 1999). Numerous researches were conducted so far with regard to elastic-core yarns, and among them several studies focused the impact of spinning parameters on the properties of elastic-core yarn. The dependence of elastane draw ratio on the tensile properties and elastic recovery of elastic core yarn were studied by many researchers and it was concluded that the tensile strength, modulus of elasticity and stretchability of core yarns increase with the increase of elastane draw ratio (Qadir et al., 2014; Babay et al., 2014; Helali et al., 2012; Su et al., 2004). Bansal et al. analyzed the quantitative influence of yarn count, twist multiplier, stretch percentage and applied extension on the tensile strength and elastic recovery behavior of the ring-spun elastane-cotton stretch yarn (Bansal et al., 2019). Elrys et al. investigated the effect of yarn structure and yarn count on the properties of different types of core-spun yarns (Elrys et al., 2021).

**Figure 1:**
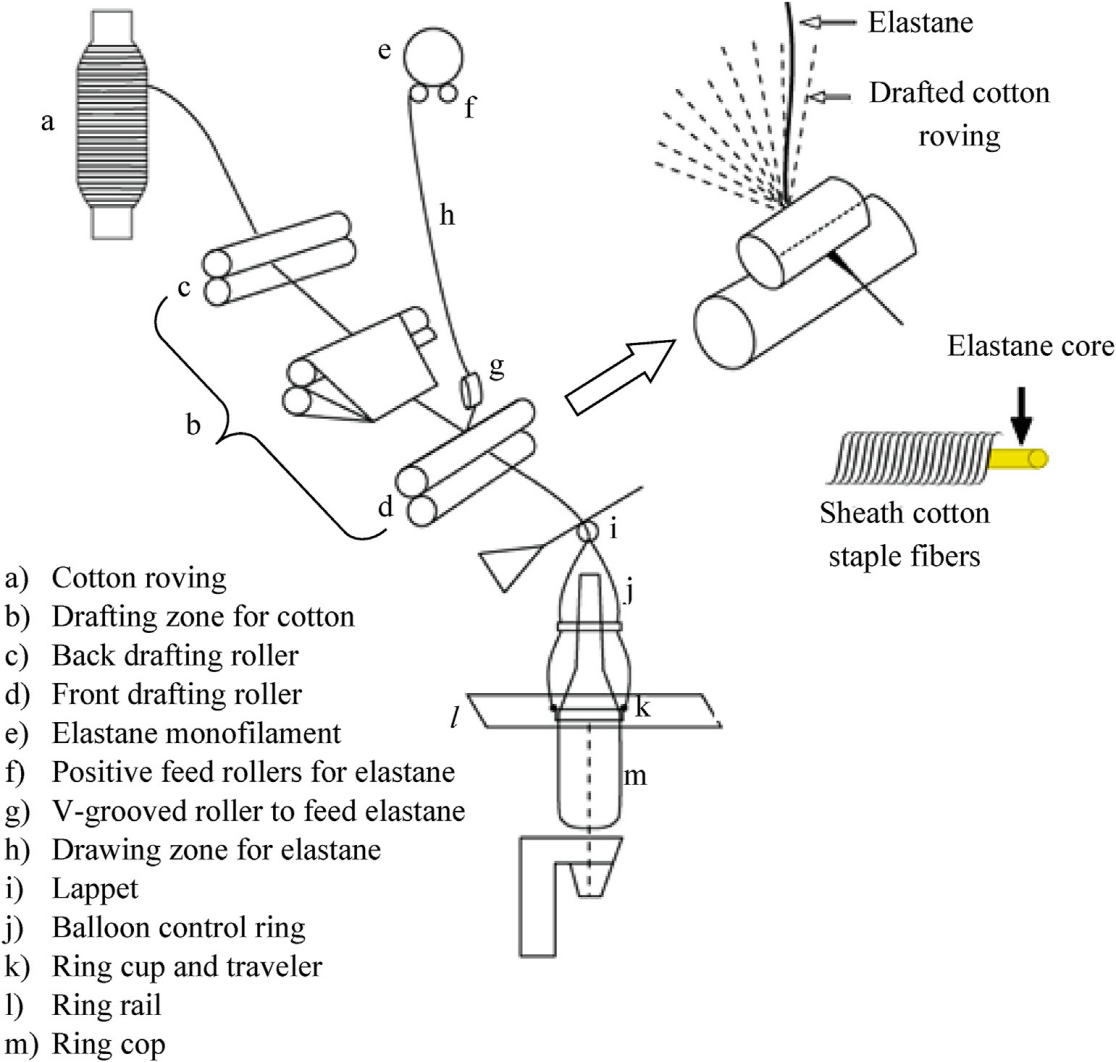
Schematic representation of conventional ring spinning frame with attachment for core elastane feeding (Adapted from Sarıoğlu and Babaarslan, 2019).

Similar to that of production of normal core yarns, the most convenient way to produce elastane core yarns is through the conventional ring spinning frame with a suitable core-feed attachment that is schematically shown in Figure 1 (Erbil et al., 2021, Sarıoğlu and Babaarslan, 2019). Here, a pair of positive feed rollers (f) feeds an elastane filament (e) to front roller (d) of ring frame via V-grooved roller (g). Cotton roving is fed to the drafting zone (b), as usual, via the back roller (c). The elastane is drawn at a certain draw ratio in between positive feed rollers (f) and front roller (d) which provides the elasticity in the core yarn (Dhouib et al., 2006).

Compact spinning is a novel concept generated through re-engineering of established ring spinning process that eliminates the spinning triangle and ensures better utilization of fibre properties in yarn leading to remarkable improvement in various yarn characteristics such as hairiness, evenness, imperfections, strength, abrasion resistance and the tendency of pilling (Basal and Oxenham, 2006; Beceren and Nergis, 2008; Liu et al., 2015). The mechanism of compacting was first tried by retrofitting a magnetic compacting mechanism in drafting zone. Later on, effectiveness of compacting performance was enhanced by installing pneumatic compacting zone in ring frame. However, the compact spinning system can be another approach for producing elastane-cotton core yarn since very few advancements of traditional ring spinning are as significant as compact spinning. A few studies on core-spun yarns manufactured with compacting mechanism were reported earlier. Barzoki et al. produced viscose/polyester and cotton covered nylon core-spun yarns using prototype RoCoS magnetic compacting mechanism where the filament pre-tension, yarn count and type of sheath fibers were found to influence the physical and mechanical properties of yarns (Barzoki et al., 2016). Dang et al., presented a new approximate approach to calculate and predict the specific stress of wool-covered spandex core-spun yarn based on an idealized structure model and Hearle's yarn theory (Dang and Wang, 2007). Zhank et al., studied the effects of godet wheel position on the characteristics of compact siro-spun core yarns (Zhang et al., 2017).

Despite having a few researches on compact-core yarns, the absence of knowledge on the quality of elastane-cotton core yarn manufactured with modern compact spinning technology highlights the need for further investigations. In this context, the current study was attempted to manufacture elastane-cotton core yarns using world's most utilized compact spinning system namely Suessen Elite compact spinning system. Suessen compact spinning is a pioneer in the compact spinning technology and a highly successful supplier of compact spinning systems. It is most versatile, flexible and can efficiently condense the fibre strand based on aerodynamic principle (EliTe® Compact Spinning System). The morphology, structure and mechanical properties of produced elastane-cotton compact core-spun yarns were compared with the elastane-cotton core-spun yarns produced in conventional ring spinning frame with the same materials and same spinning parameters.

## 2 Materials and methods

### 2.1 Materials

Cotton fibers (blend of Chad-42.54%, Brazil-30.35%, Organic India-15.29% and USA-11.82%) with mean fineness of 4.49 micronaire value, 2.5% span length of 29.28 mm and tenacity of 29.35 g/tex were used as sheath fibers. Creora (Vietnam) of 78 dtex monofilament was used as core elastane. Raw cotton properties tested through HVI (High Volume Instrument) & AFIS (Advanced Fiber Information System) are shown in Table 1 and Table 2.

**Table 1:** Raw cotton specifications (HVI).

Raw Cotton	SCI	Micronaire	Length (mm)	Tenacity (g/tex)	Elongation (%)
Brazil	121	4.17	27.63	29.4	6.3
USA	120	4.42	29.56	28.7	6.5
Chad	124	4.53	29.67	28.2	5.9
Organic India	135	4.84	30.27	31.0	8.0

**Table 2:** Raw cotton specifications (AFIS).

Raw Cotton	Neps (cnt/g)	SCN(cnt/g)	SFC(w)	SFC(n)	IFC (%)
Brazil	321	20	9.1	25.5	9.2
USA	464	19	10.9	28.5	8.7
Chad	235	20	6.2	19.3	9.7
Organic India	105	17	8.3	23.9	7.6

### 2.2 Methods

#### 2.2.1 Preparation of roving from raw materials

In this work, elastane core yarns were produced following the carded process. The detail of the machines and technical parameters to produce roving from raw cotton are given in Table 3.

**Table 3:** Production details for preparation roving from raw materials.

Process	Machine & Model	Delivery material	Speed
Blow Room	Trutzschler	550 ktex card mat	Chute feed to card
Carding	Rieter C-70	5.7 ktex card sliver	200 m/min
Breaker Draw Frame	Rieter SB-D-30	4.96 ktex drawn sliver	700 m/min (doubling 8)
Finisher Draw Frame	Rieter SB-D-45 (with autoleveller)	4.96 ktex drawn sliver	700 m/min (doubling 8)
Simplex	Toyoda FL-100	0.8 ktex roving (TPI-1.05)	900 rpm (Flyer speed)

#### 2.2.2 Production of elastane-cotton core yarn by the conventional ring spinning system

**Figure 2:**
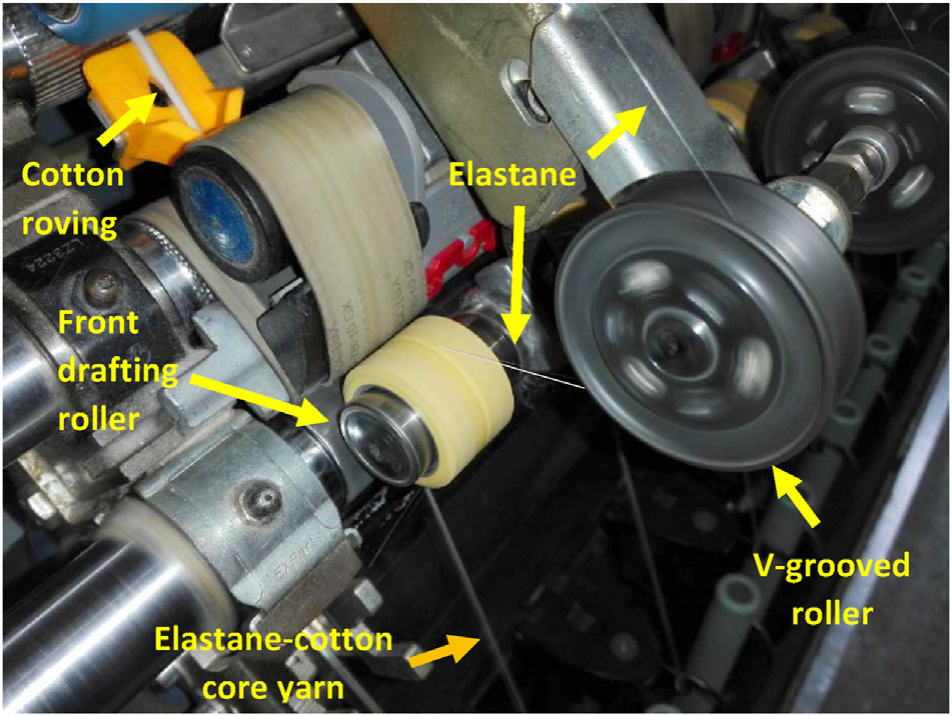
Manufacturing of elastane-cotton core yarn on the conventional ring spinning frame.

Marzoli DTM 129 ring frame with Amsler core yarn attachment was used to manufacture elastane-cotton core yarn by the conventional ring spinning system (Figure 2). Amsler is one of the market leaders nowadays for its core yarn attachment in the world. The system of the attachment simplifies the handling along with keeping and improving the elastane centering performance as well as the rock-solid filament monitoring and roving stop function (Amsler core spinning attachment, 2019).

The 0.8 ktex cotton roving was fed through the back roller of the ring frame. A 78 dtex elastane package mounted on positive feed rollers was passed forward via V-grooved roller and combined with the drafted roving to the back of front top roller (indicated in Figure 2). By rotating the control key of the core attachment, the elastane draw ratio was adjusted at 3.8. Many spinning mills in Bangladesh practice draw ratio 3.8 for creora 78 dtex elastane to produce elastane core yarns on the basis of better tensile properties and elastic recovery. In this way, weaving grade elastane-cotton core yarns with different counts viz. 20 tex (TM: 4.9), 30 tex (TM: 5.0) & 60 tex (TM: 5.8) with 11,500 spindle speed were manufactured. Core yarns with this count range are commercially manufactured to produce light to heavy weight denim fabrics.

#### 2.2.3 Production of elastane-cotton core yarn by the compact spinning system

**Figure 3:**
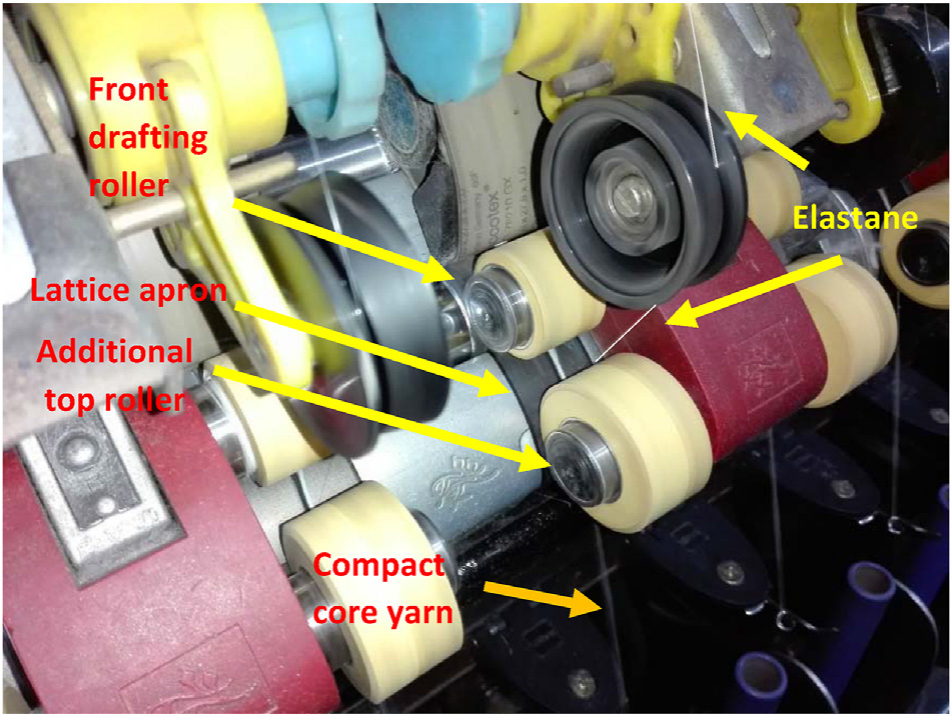
Manufacturing of elastane-cotton core yarn on the compact spinning system.

Twelve units (6 spindles per unit × 2) of Suessen Elite® compact attachment were experimentally installed on the same Marzoli DTM 129 ring frame machine those were used to manufacture elastic core yarn with conventional method. In Suessen Elite® compacting system the condensation of the fiber strand takes place aerodynamically with the help of a profile tube that is closely embraced by a perforated lattice apron driven by an additional top roller (Lawrence, 2010). The elastane is combined with the drafted fiber strand at the back of the additional top roller (shown in Figure 3). Again, similar three counts of yarns of 20 tex, 30 tex & 60 tex elastane-cotton core yarns were manufactured using the compact spinning units with same materials and same machine parameters.

### 2.3 Measurements

Coefficient of mass variation (CVm%), imperfection, and hairiness were measured by USTER® Tester 5 (UT5), model S400 in accordance with ASTM D1425/1425M-14 (2020) standard test method. For evenness testing, 25 m sliver was tested at testing speed 25 m/min, 50 m roving was tested at testing speed 50 m/min and 400 m yarn sample was tested at speed of 400 m/min.

Yarns spun from staple fibers contain extreme of variations, referred as imperfections. The imperfections are subdivided into three categories: thin places, thick places and neps. In this study, the sensitivity thresholds of thick place (+50%), thin place (−30%), and neps (+200%) per 1000 m of yarn were used. Yarn hairiness (H), measured by the hairiness sensor of evenness tester by optical principle, is the total length of protruding fibers (in cm) within the measurement field of 1 cm length of yarn.

Single yarn strength and elongation were ascertained by Titan Universal Testing Machine, James Heal under CRE (constant rate of extension) principle according to ASTM D 2256 standard. All the experiments were accomplished after conditioning the samples in standard testing atmosphere (65 ± 2% RH and 20 ± 2 °C) for 24 h. 100 N load cell, 250 mm gauge length, 250 mm/min a crosshead speed and 5 cN pretension were used. The experimental results represent the average of 10 individual measurements.

The surface morphology of the yarns was investigated using scanning electron microscope (SEM) images using SEM, JEOL 6460LV, Tokyo, Japan. The accelerating voltage was 5.0 kV. Finally, the correlation and significance of yarn count with various quality parameters of yarns were checked by using IBM SPSS 25 software.

## 3 Results and discussion

### 3.1 Unevenness of sliver and roving

**Figure 4:**
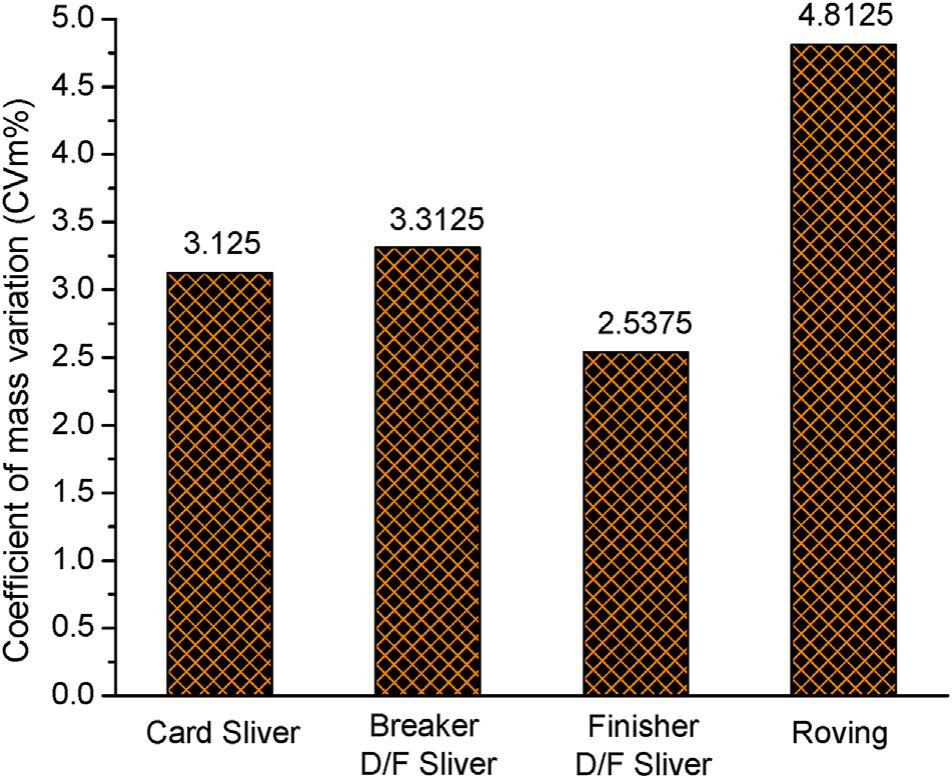
Coefficient of mass variation (CVm%) of card sliver, breaker D/F sliver, finisher D/F sliver and roving.

Unevenness, usually expressed by coefficient of mass variation (CVm%), of card sliver, breaker draw frame (D/F) sliver, finisher D/F sliver and roving are shown in Figure 4. Compared with card sliver, CVm% of breaker D/F sliver slightly increased as 5.7 ktex card sliver was drafted to make thinner 4.96 ktex sliver. Though 8 card slivers were doubled in breaker D/F, unevenness imposed by the applied draft by the drafting elements predominates over the reduction of unevenness by equalizing action through doubling. Since the count of breaker D/F and finisher D/F sliver was same i.e. 4.96 ktex, the CVm% of finisher D/F sliver significantly decreased because of doubling effect, lower draft than breaker D/F and levelling action of autoleveller. CVm% of roving markedly increased as there was no doubling here and draft 6.2 was imposed on a single finisher D/F sliver to convert it into roving.

### 3.2 Structural aspects of yarns

**Figure 5:**
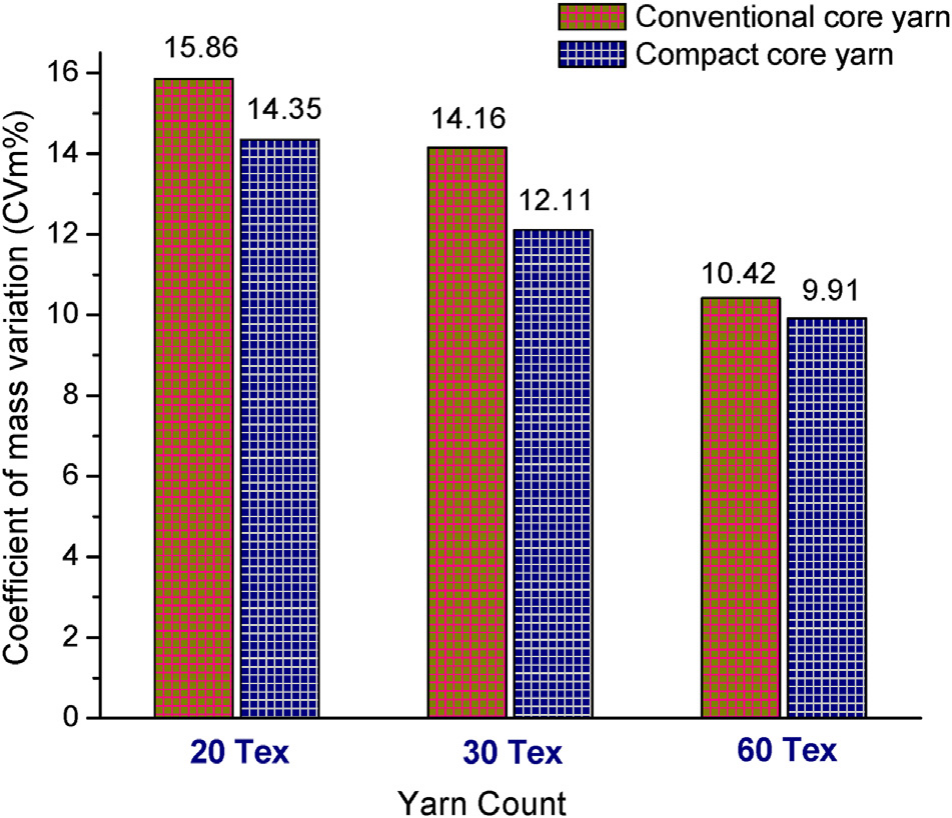
Co-efficient of mass variation (CVm%) of different counts of core yarns produced in conventional and compact spinning systems.

Unevenness or coefficient of mass variation (CVm%) of elastane-cotton core yarn samples of different counts such as 20 tex, 30 tex and 60 tex produced in conventional and compact spinning system are illustrated in Figure 5. As seen in Figure 5, CVm% of yarns decreases with the increase of yarn coarseness i.e. tex. The reason is ascribed primarily to the application of lower draft for coarser yarns as it is well known that draft causes irregularity. In addition, the presence of higher number of fibers in the cross-section of coarser yarn causes equalizing effect resulting in lower CVm% values. However, Figure 5 illustrates that CVm% are lower for all compact core yarns than those of conventional core yarns. The reason may be interpreted as follows.

In conventional ring spinning, the spinning triangle appears just after emerging the fiber strand from front roller nip because of higher width of the drafted fiber ribbon as compared to final yarn diameter. The fibers in the spinning triangle are subjected to varying tensions depending upon their positions and edge fibers of spinning triangle are strongly deflected and cannot be evenly integrated with yarn body, some escape the twist partially and some are lost as fly. Resultantly full utilization of fiber length cannot be achieved in the yarn and many fibers show up partly as protruding hairs in yarn surface. The edge fibers do little or no contribution to the yarn strength (Kumar, 2014). Conversely, the mechanism of compact spinning involves the elimination of the spinning triangle by narrowing the width of the fiber ribbon which come out from the final drafting rollers before it is twisted into a yarn. The compact yarn technology thus allows more parallelization and condensation of the fibers in condensing zone after the main draft that ensures incorporation of most of the fibers in the yarn body. This ultimately results in reduction in yarn unevenness (Soydan et al., 2019). Not only low unevenness, the mechanism of compact spinning also results in dramatic reduction in hairiness of yarns (Haleem et al., 2019).

The spun yarns, in principle, are manufactured by twisting the staple fibers where protruding fibers appear as fuzziness or hairiness in yarn surface (Umair et al., 2017a). Carded yarns are more hairy compared to combed yarns as no short fibers are removed by employing combing process (Umair et al., 2017b). Excessive hairiness affects the yarn performance in weaving and knitting, and hairiness variation can downgrade certain end products such as the appearance and feel of the fabric. Moreover, variation in hairiness is a source of weft bars and warp way streaks in the fabric (Thilagavathi and Karthik, 2016).

**Figure 6:**
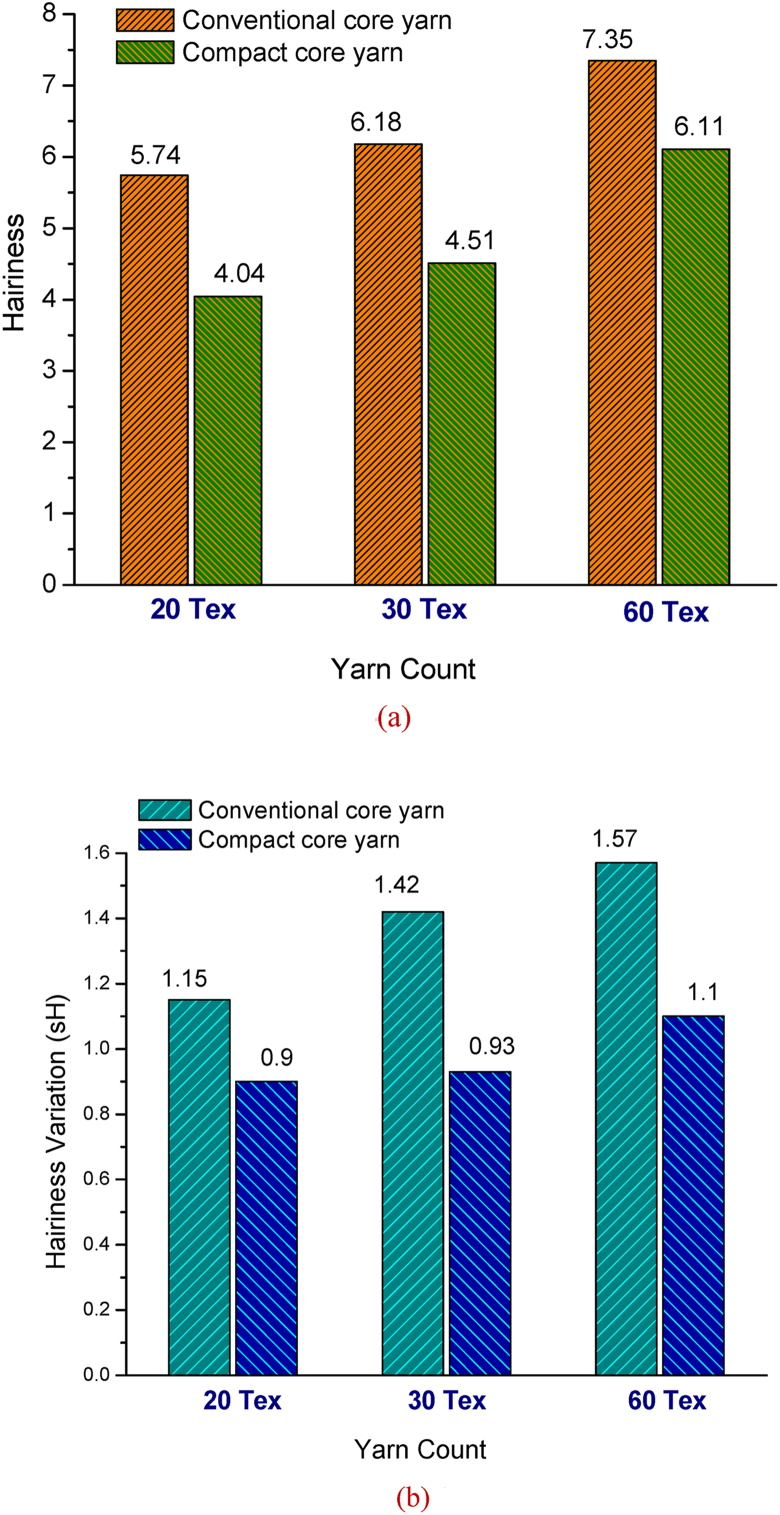
(a) Hairiness of different counts of core yarns produced in conventional and compact spinning systems. (b) Hairiness variation (sH) of different counts of elastane-cotton core yarns produced in conventional and compact spinning systems.

Figure 6(a) depicts the hairiness values of different counts of elastic core yarns produced in conventional and compact spinning systems. Here, it is visible that hairiness of yarns increases with the increase of yarn coarseness i.e. tex. This is due to the higher number of fibers in the yarn cross section. Moreover, coarser yarn needs lower twist which is also responsible for higher hairiness values. However, the hairiness values significantly decreased for all compact core yarns. For example, in case of 20 tex yarn, the hairiness value 5.74 of conventional core yarn decreases to 4.04 for compact core yarn that is approximately 30% lower. Similarly, remarkable decrease in hairiness is also observed for 30 tex and 60 tex yarns.

Figure 6(b) shows the hairiness variation (sH) of yarns. The standard deviation sH (cut length 1 cm) is a measure for the variation of the yarn hairiness. As shown in Figure 6(b), core yarns produced in compact spinning exhibit remarkably lower sH values.

Hairiness value of elastane-cotton core yarns is usually much higher than the normal cotton yarn as sheath fibers cannot bind into the yarn body properly due to uncontrolled movement of sheath fibers during spinning (Das and Chakraborty, 2013). The findings obtained from Figure 6(a) and Figure 6(b) suggest that by employing compact spinning system, both hairiness and hairiness variation of elastane-cotton core yarns can be reduced to a great extent.

**Figure 7:**
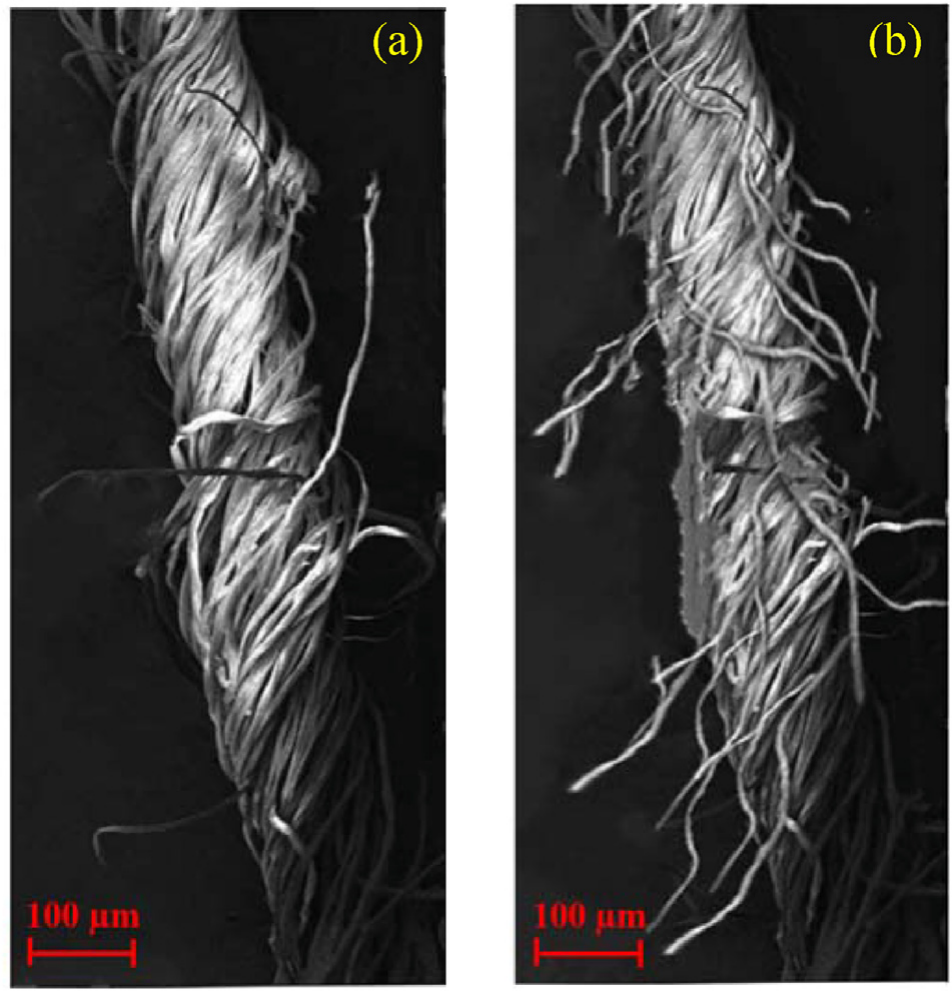
SEM images of 20 tex elastane-cotton core yarns produced in (a) compact and (b) conventional ring spinning.

The reduction of hairiness of elastane compact core yarn can be substantiated by studying the surface morphology of yarns through scanning electron microscopy (SEM). As exhibited the SEM images in Figure 7, obvious differences in hairiness of two elastic core yarns produced in compact and conventional ring frame are discernible. As shown in Figure 7(a), elastic compact core yarn shows a very dense structure with highly parallel fibers and much less hairiness. As explained above, in compacting mechanism twist is imparted to a straightened and optimally condensed fiber strand with individual and parallel fibers without protruding hairs. Such low hairiness of compact core yarns is expected to bring considerable improvement in further processing such as reduction in clinging tendency of yarns during shed formation. In addition, such yarns will require less size (30–35%) during sizing for weaving yarn and less waxing for knitted yarns. The low degree of yarn hairiness may eliminate the singeing process and reduce the consumption of dyestuffs in dying process (Jackowski et al., 2004). The products produced from such yarns also provide smooth and lustrous fabric surface with high abrasion and pilling resistance (Compact spinning K 47 of Rieter, Application Manual). On the other hand, elastic core yarn produced in conventional ring spinning, as shown in Figure 7(b), exhibits far less perfect structure than compact yarn. Many fibers are attached to the yarn in a disorderly configuration. The unincorporated edge fibers in spinning triangle stand out from the twisted yarn core, and cause long hairiness on the yarn surface. Such protruding fibers give a rough feel to the yarn as well as the products and are the reason of high fly generation along with a number of problems in downstream processing. For instance, long protruding hairs from the yarn contribute to multiple breaks in weaving and fabric faults like stitches and floats (Khurshid et al., 2013).

**Figure 8:**
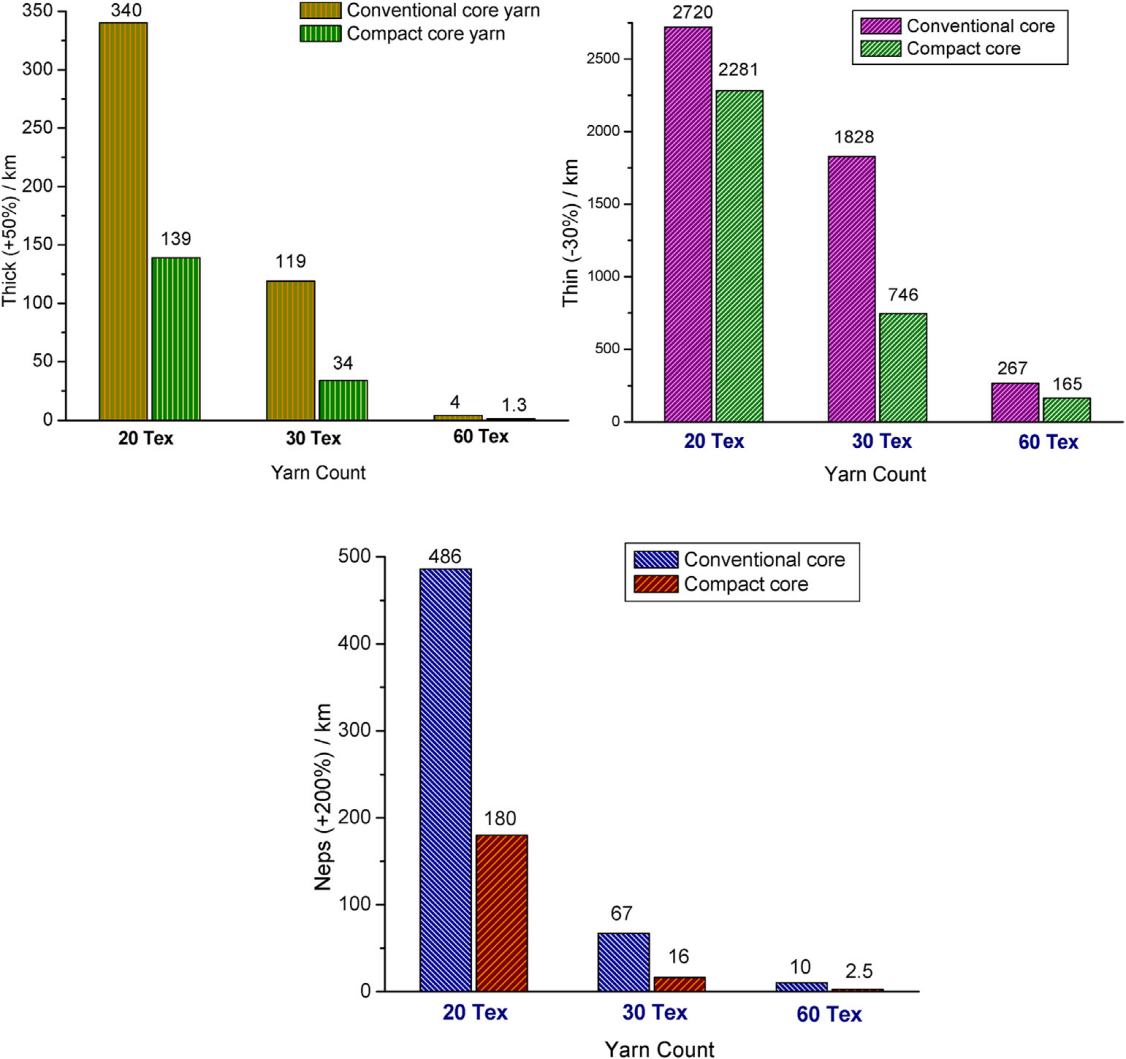
Imperfections of different counts of elastane-cotton core yarns produced in conventional and compact spinning systems: (a) thick places, (b) thin places, and (c) neps.

Yarns with more imperfections viz. thick places, thin places and neps can perform poorly in a later processes, e.g., they can lead to end-down in the warping, sizing, weaving and knitting processes. Imperfections can also quite considerably affect the appearance of a woven or knit fabric (Regar et al., 2017). The imperfections i.e., thick place (+50%), thin place (−30%), and neps (+200%) of elastic core yarns produced in conventional and compact spinning are exhibted in Figure 8(a), (b) and (c), respectively. Compared with conventional core yarn, significant decrease in thick, thin and neps is marked for the compact core yarn. In compact spinning system, fibers are well-controlled in the condensing zone assisted by suction that gives a firmer yarn body. The suction system also helps the protruding fibers to get integrated into the yarn structure. Well-controlled fiber strand with additionally integrated fibers improve the consolidation of yarn structure that contribute to reduce the thick and thin places of yarn (Dou and Liu, 2011).

Neps, consisted of chiefly small cluster of entangled fibers and foreign matters, are considered as blemishes in dyed fabrics. The severity of neps may even be a reason of lot rejection after final stage of garments production. Neps can be reduced by regular monitoring of all intermediate spinning processes, especially carding and combing, and the complete avoidance of neps during the production of spun yarns is a fundamental technological limitation. Compacting zone in spinning was previously reported to reduce the unevenness, hairiness and total imperfection (sum of thick, thin and neps values) of yarns (Khurshid et al., 2013). It is generally known that compacting mechanism cannot contribute in reduction or elimination of neps. In contrary to the previous reports, a fascinating outcome of neps reduction for the elastic compact core yarns is reported in this work that is demonstrated in Figure 8(c). The reason for the dramatic reduction of neps value in case of elastic compact core yarns may be ascribed to the following fact.

**Figure 9:**
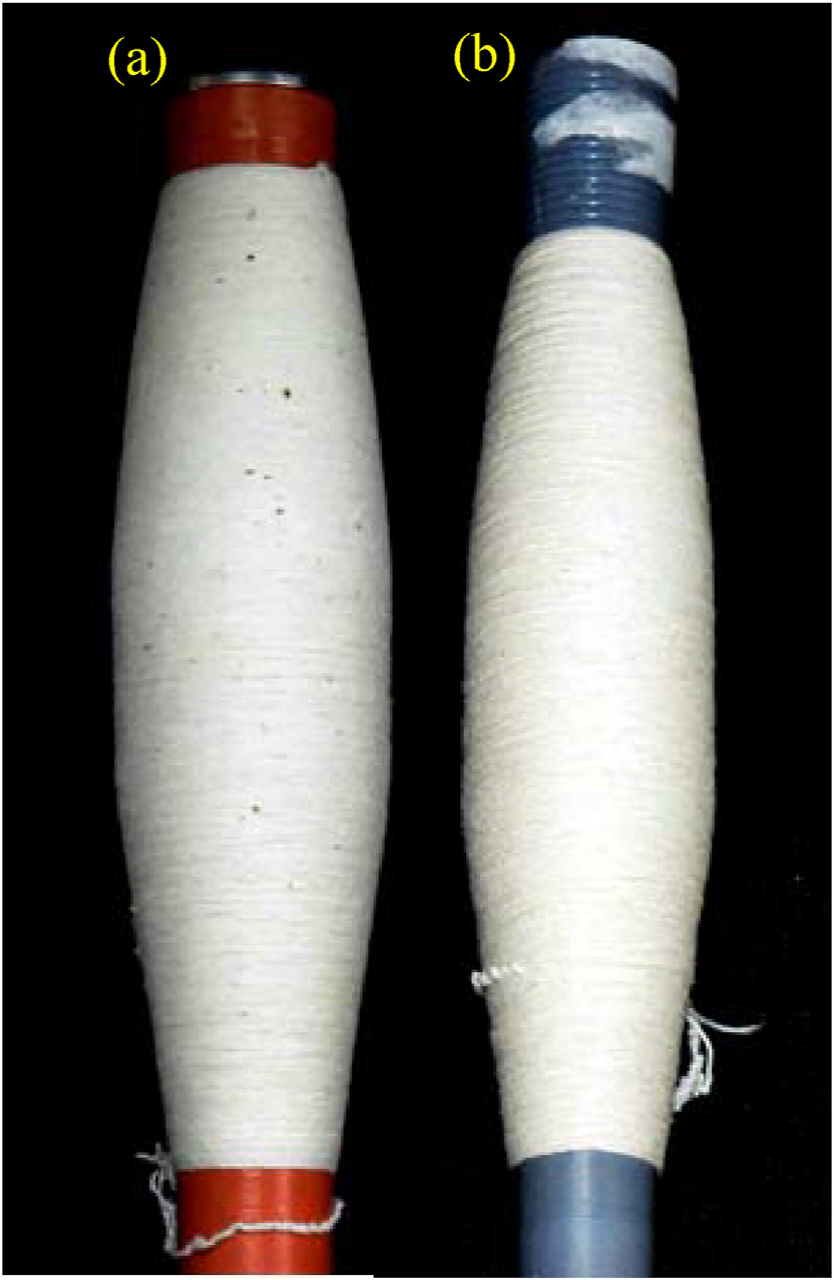
Images of 30 tex elastane-cotton core yarn ring cops produced in (a) conventional and (b) compact spinning systems.

When straightened and parallel fiber strand of compacting zone along with elastane goes through the twisting operation, many neps, mostly seed-coat neps (SCNs), could presumably not integrate with the yarn body. Being bigger and heavier in sizes, SCNs might have been separated from the fibers by the pneumatic suction system of compacting zone and thrown away from the yarn body, in presence of core elastane, owing to centrifugal force during the ballooning action. This interpretation can be evidenced from the visual observations of yarns as shown in Figure 9. As it is seen, the elastic core yarn produced in conventional spinning retains quite a large number of SCNs compared with the elastic core yarn produced in compact spinning.

### 3.3 Correlation of yarn structure with mechanical properties

The impact of lower unevenness (CVm%) and imperfections of elastic compact core yarns on the tensile properties, such as tenacity and elongation, were studied. The tenacity of a yarn is its breaking force per linear density and it is an important property required in fabric manufacturing process especially in high-speed weaving where the yarn is withdrawn with high stress imposed by various machine elements under dynamic condition (Kumar et al., 2003). More specifically, the yarn tenacity heavily influences the end breakage rate as well as the efficiency in spinning, winding, weaving, sizing and knitting processes (Jackowski et al., 2002). When load is imposed to a yarn, it is distributed among the component fibers and on account of this, elongation is another significant property of yarn. The elongation of a yarn is related to respective fiber extension, and the alignment and cohesion among fibers in yarn structure (Almetwally et al., 2015).

**Figure 10:**
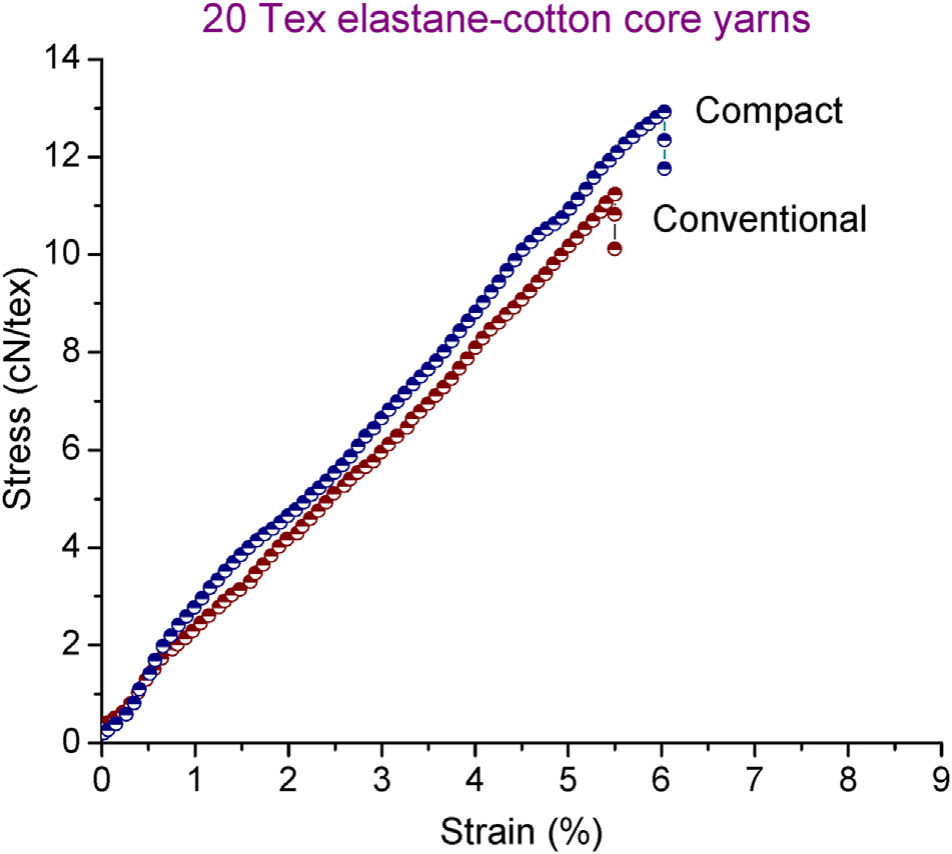
Representative stress-strain curves of 20 tex elastane-cotton core yarn produced in conventional and compact spinning systems.

**Figure 11:**
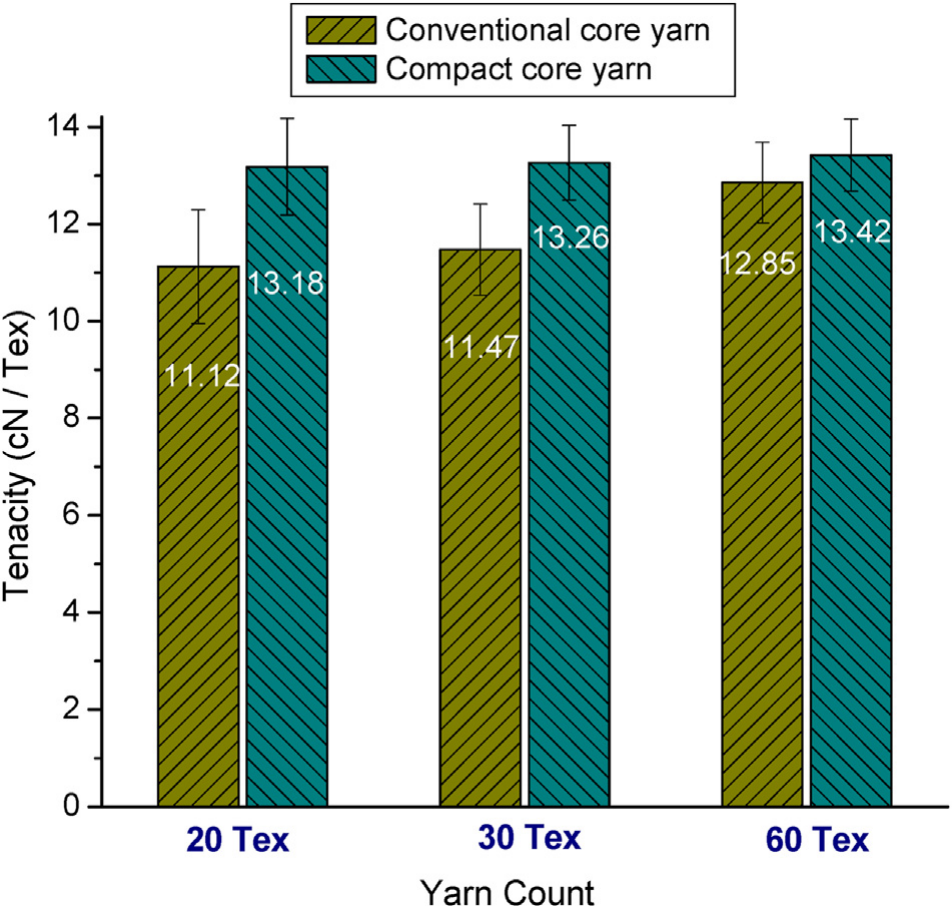
Tenacity of different counts of elastic core yarns produced in conventional and compact spinning systems.

**Figure 12:**
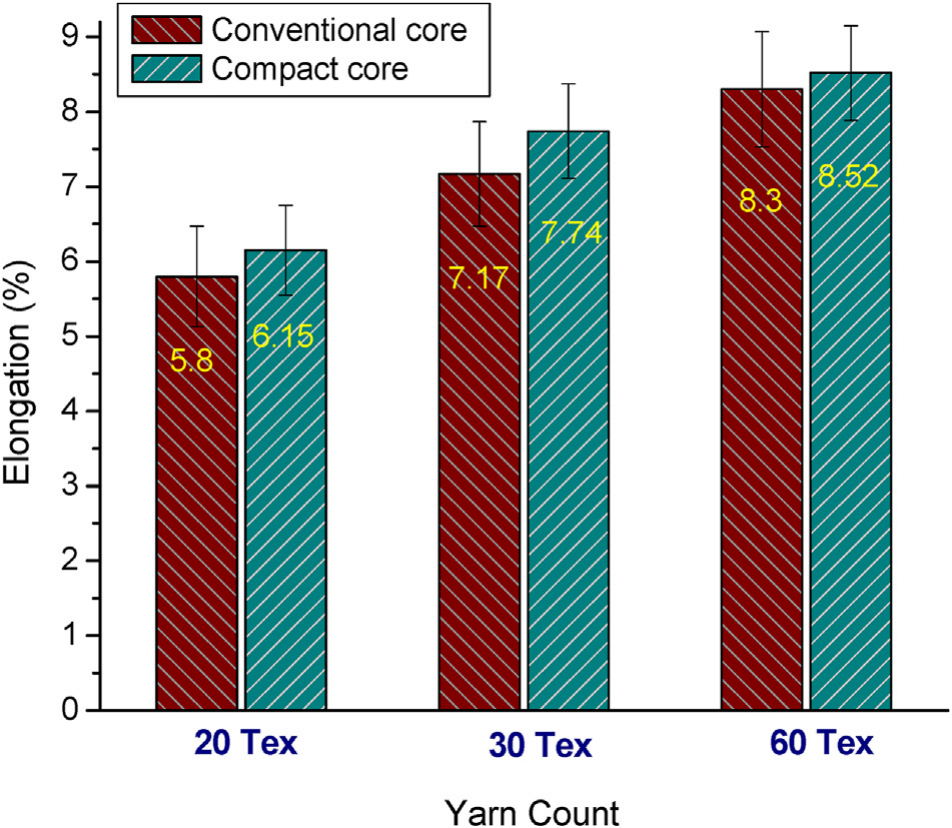
Elongation at break% of different counts of elastic core yarns produced in conventional and compact spinning systems.

Figure 10 shows the representative stress-strain curves of 20 tex elastane-cotton core yarn produced in conventional and compact spinning systems. Similar patterns were found for 30 tex and 60 tex yarns also (not shown here). The shapes of the stress-strain curve of the elastic compact core yarns were almost linear and similar to that of elastic core yarn produced in conventional ring spinning system. However, the slope and the end point are higher for the elastic compact core samples indicating that compact core yarns attained higher strength and elongation. Tenacity and elongation at break%, evaluated from the respective stress-strain curves, are shown in Figure 11 and Figure 12, respectively. As seen in Figure 11, tenacity of all elastic compact core yarns is found to be increased reflecting the corresponding decrease in unevenness and imperfection values (shown before in Figure 5 and Figure 8). Higher breaking elongation for compact core yarns were also observed as shown in Figure 12. The higher tenacity and elongation of compact core yarns can be attributed to the fact that compacting zone with the help of air suction aids in the integration of projecting fibers into the yarn structure. Yarn uniformity and consolidation develop due to such fiber integration led the yarn to higher strength. Improved integration and alignment of fibers in the surface as well as the firm structure of compact core yarn resulted in corresponding increment in strength and elongation. Similar outcomes were previously reported for normal compact yarns (Wu et al., 2009; Wei et al., 2016; Regar et al., 2017).

### 3.4 Statistical analysis

The correlations and significance of paired samples such as yarn count of compact and conventional core yarns with various yarn parameters are shown in Table 4. As seen in Table 4, a negative correlation was found between compact core yarn count and yarn unevenness (−0.873) i.e., with the increase of compact yarn count (Tex), yarn unevenness decreased (seen before in Figure 5). Negative correlations were also found for thin place (−0.816), thick place (−0.784) and neps (−0.733). On the other hand, with the increase of compact yarn count (Tex), the hairiness, tenacity and elongation values increased that are substantiated by the positive correlation values such as 0.809, 0.797 and 0.712, respectively. All the relations are found to be highly significant (p = 0.000).

**Table 4:** Paired samples correlations for compact and conventional elastic core yarns.

Variables	Compact elastic core yarn		Conventional elastic core yarn	
	Correlation coefficient	Significance	Correlation coefficient	Significance
Yarn count & Unevenness	-0.873	0.000	-0.937	0.000
Yarn count & Thin	-0.816	0.000	-0.977	0.000
Yarn count & Thick	-0.784	0.000	-0.853	0.000
Yarn count & Neps	-0.733	0.000	-0.743	0.000
Yarn count & Hairiness	0.809	0.000	0.839	0.000
Yarn count & Tenacity	0.797	0.000	0.774	0.000
Yarn count & Elongation	0.712	0.000	0.821	0.000

Similar correlation and significance were obtained for the conventional yarn counts with various yarn parameters.

## 4 Conclusion

Elastane-cotton core yarns with different counts (20 tex, 30 tex and 60 tex) were successfully manufactured in most modern pneumatic compact spinning system, and the structure and properties were thoroughly investigated. Compared with the core yarns produced in conventional spinning system, the yarns manufactured in compact spinning showed noticeably lower unevenness, imperfections, hairiness and hairiness variation which are closely related to the processing of yarn in later processes and aesthetics of the fabric as well. The improved structural parameters of compact core yarns contributed in attaining higher tenacity and elongation values which are essential for both weaving and knitting. Most striking outcome of this study is the drastic reduction of neps, mostly seed-coat neps, by the presence of elastane core in compact spinning system.

Based on the overall outcome of this study, it is concluded that compact spinning system, revolutionary version of ring spinning, can be utilized to produce elastane-cotton core yarns with good structural parameters and improved mechanical properties.

## Declarations

### Author contribution statement

Md. Ikramul Islam: Conceived and designed the experiments; Performed the experiments; Contributed reagents, materials, analysis tools or data; Wrote the paper.

Ahmed Jalal Uddin: Conceived and designed the experiments; Analyzed and interpreted the data; Contributed reagents, materials, analysis tools or data; Wrote the paper.

### Funding statement

This research did not receive any specific grant from funding agencies in the public, commercial, or not-for-profit sectors.

### Data availability statement

No data was used for the research described in the article.

### Declaration of interests statement

The authors declare no conflict of interest.

### Additional information

No additional information is available for this paper.
